# The Role of Psychobiotics in Supporting the Treatment of Disturbances in the Functioning of the Nervous System—A Systematic Review

**DOI:** 10.3390/ijms23147820

**Published:** 2022-07-15

**Authors:** Krzysztof Skowron, Anna Budzyńska, Natalia Wiktorczyk-Kapischke, Karolina Chomacka, Katarzyna Grudlewska-Buda, Monika Wilk, Ewa Wałecka-Zacharska, Małgorzata Andrzejewska, Eugenia Gospodarek-Komkowska

**Affiliations:** 1 Department of Microbiology, Ludwik Rydygier Collegium Medicum in Bydgoszcz, Nicolaus Copernicus University in Toruń, 85-067 Bydgoszcz, Poland; a.budzynska@cm.umk.pl (A.B.); natalia12127@gmail.com (N.W.-K.); ka.chomacka@gmail.com (K.C.); katinkag@gazeta.pl (K.G.-B.); monikawilk7878@gmail.com (M.W.); gospodareke@cm.umk.pl (E.G.-K.); 2 Department of Food Hygiene and Consumer Health, Wrocław University of Environmental and Life Sciences, 50-375 Wrocław, Poland; ewa.walecka@upwr.edu.pl; 3 Department of Hygiene, Epidemiology, Ergonomy and Postgraduate Education, Ludwik Rydygier Collegium Medicum in Bydgoszcz, Nicolaus Copernicus University in Toruń, 85-067 Bydgoszcz, Poland; m.andrzejewska@cm.umk.pl

**Keywords:** psychobiotics, gut–brain axis, microbiome, probiotics, nervous system diseases, neurodegenerative diseases, depression, autism spectrum disorders, Alzheimer’s disease, Parkinson’s disease

## Abstract

Stress and anxiety are common phenomena that contribute to many nervous system dysfunctions. More and more research has been focusing on the importance of the gut–brain axis in the course and treatment of many diseases, including nervous system disorders. This review aims to present current knowledge on the influence of psychobiotics on the gut–brain axis based on selected diseases, i.e., Alzheimer’s disease, Parkinson’s disease, depression, and autism spectrum disorders. Analyses of the available research results have shown that selected probiotic bacteria affect the gut–brain axis in healthy people and people with selected diseases. Furthermore, supplementation with probiotic bacteria can decrease depressive symptoms. There is no doubt that proper supplementation improves the well-being of patients. Therefore, it can be concluded that the intestinal microbiota play a relevant role in disorders of the nervous system. The microbiota–gut–brain axis may represent a new target in the prevention and treatment of neuropsychiatric disorders. However, this topic needs more research. Such research could help find effective treatments via the modulation of the intestinal microbiome.

## 1. Introduction

Emotional well-being is a relevant component of human health at any age. Modern society results in stress or anxiety almost every day. Many people in the United States suffer from mental illness every year, with health and financial consequences [1]. In recent years, there has been an increased interest among the medical community in the gut–brain axis, its functioning, and its influence on individual disease entities. Recently, research has been conducted on the impact of probiotics on the cerebral and intestinal axis and the health of patients with mental disorders [2].

A key aspect of maintaining mental health is the two-way communication between the intestine and the brain [3]. The latest reports inform about the direct or indirect influence of the microbiome on anxiety and depressive disorders. Researchers have shown the link between intestinal dysbiosis and autism spectrum disorders and neurodegenerative diseases, such as Parkinson’s disease and Alzheimer’s disease [3,4].

### 1.1. Psychobiotics

**Figure 1:**
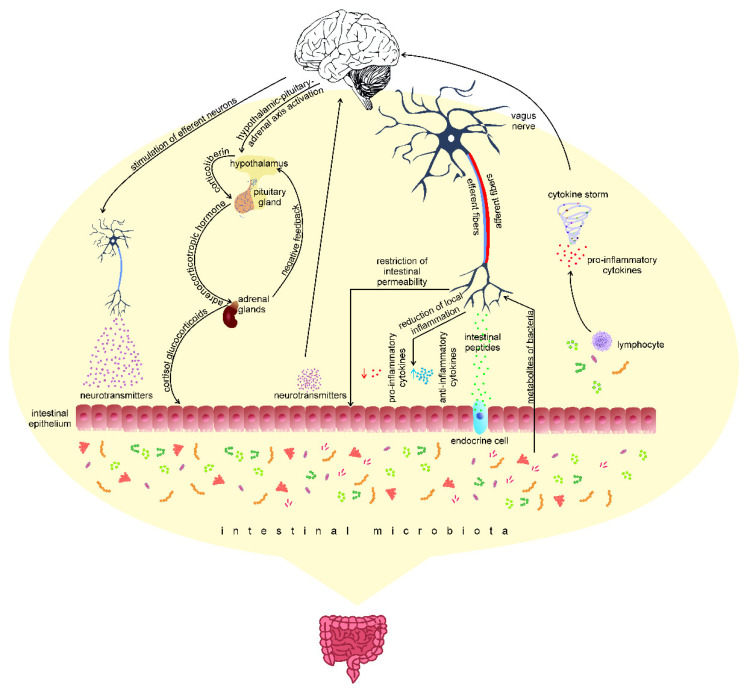
Central nervous system stimulation as part of the functioning of the gut–brain axis may occur through 3 afferent pathways: (1) production of endocrine or paracrine cytokines by lymphocytes in contact with the microbiota, (2) activation of neuron terminals by intestinal peptides secreted by enteroendocrine cells, and (3) exerting an endocrine or paracrine effect in intestinal epithelial cells by neurotransmitters or their precursors produced by the intestinal microbiota. After the activation of the central nervous system, the signal reaches the brain stem (e.g., the nucleus of the solitary strand), and it is then transferred to a separate neural network consisting of the amygdala and the cortex of the island, whose task is to integrate information from internal organs. In response to the signal, the activation of the hypothalamic–pituitary axis of the adrenal gland and the secretion of corticosteroids, as well as the stimulation of efferent neurons, may occur, resulting in the activation of the cholinergic anti-inflammatory pathway and/or the sympathetic nervous system, causing the release of classical neurotransmitters, according to [15,16,17].

Probiotics, as defined by the Food and Agriculture Organization of the United Nations (FAO) and the World Health Organization (WHO), are live microorganisms that, when administered in appropriate amounts (approximately 1 × 10^9^ cells/day), have a beneficial impact on the host organism [5]. Probiotics are most often used to support treatment, especially during antibiotic therapy and/or immediately after completion. This therapy aims to restore the balance of the intestinal microbiome disturbed after drug therapy [6]. The psychiatrist Ted Dinan and neuroscientist John F. Cryan introduced a new term for probiotics that positively affect the health of patients with mental disorders. They called them psychobiotics [7]. Psychobiotics differ from probiotics in their ability to produce or stimulate the production of neurotransmitters, short-chain fatty acids, enteroendocrine hormones, and anti-inflammatory cytokines [3]. The effectiveness of psychobiotics is attributed to their influence on the microbiota–gut–brain axis. This axis is a continuous two-way communication pathway between the gut microbiome and the central nervous system [8,9]. The efferent pathway of the central nervous system is closely related to maintaining homeostasis in the body [10,11] and the functioning of the intestines—it controls, among others, gastrointestinal peristalsis and blood supply [10]. The microbiota may influence the functioning of the nervous system and the pathogenesis and development of diseases related to the nervous system [9,11]. Both elements of the axis are connected, among others, through the intestinal nervous system, the vagus nerve (X), the immune system, and the endocrine and metabolic pathways (Figure 1) [12]. Microorganisms that inhabit the intestines (intestinal microbiota), including bacteria, fungi, viruses, and protozoa, participate directly or indirectly in all connections of the cerebral–intestinal axis [9]. Disturbances in the composition and quantity of intestinal microorganisms can affect both the intestinal nervous system and the central nervous system (CNS) [13]. Studies have shown that intestinal microorganism metabolism can regulate microglia maturation and functioning, thus influencing CNS functioning [14].

#### 1.1.1. Microorganisms and Their Metabolites

Scientists have not fully elucidated how bacteria exploit their psychobiotic potential. Psychobiotic potential can be achieved in three ways: (1) by stress response to the hypothalamic–pituitary–adrenal (HPA) axis and reduction in systemic inflammation; (2) by a direct influence on the immune system; and (3) through metabolites (neurotransmitters, proteins, and short-chain fatty acids (SCFAs)) [18].

The most important metabolites of microorganisms are SCFAs, neuroactive substances, short peptides, and microbial toxins. These substances interact directly with the appropriate receptors present in enteroendocrine cells, in the vagus nerve, or move through the intestinal epithelium to the peripheral circulation. SCFAs (e.g., acetate, propionate, and butyrate) are one of the predominant metabolites of bacterial fermentation in the colon and small intestine. They are responsible for the regulation of many physiological processes, such as histone acetylation (acetate), the induction of T cells in the colon (butyrate), glucose and cholesterol metabolism in various tissues, and adipolysis (acetate and propionate) [19]. In addition, SCFAs control the release of hormones, such as the YY peptide (PYY), glycogen-like protein (GLP-1), and cholecystokinin (CCK) [20].

The gut microbiota react to the presence of neurohormones in the environment and also produce them (serotonin, dopamine, and noradrenaline) [21,22]. Interestingly, almost half of the dopamine in the human body is produced by microorganisms that inhabit the gastrointestinal tract [23]. The gut microbiota also contribute to the production of stress hormones. Table 1 presents the general functions of neurotransmitters.

#### 1.1.2. The Intestinal Microbiome and Disorders of the Nervous System

**Figure 2:**
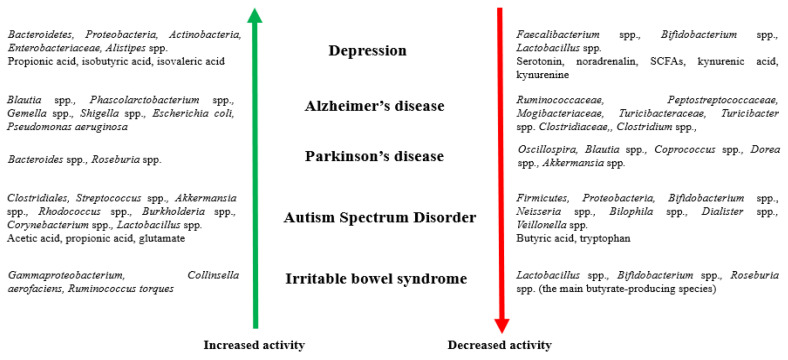
Participation of microorganisms and their metabolites (increased and decreased activity) in disorders of the nervous system [34,35,36,37,38,39,40,41,42,43,44,45,46].

Disturbances to the gut microbiome can contribute to an abnormal stress response and affect the communication of the microbiome–gut–brain axis, leading to the development of nervous system disorders [33]. Figure 2 presents the participation of microorganisms and their metabolites in disorders of the nervous system.

New possibilities in the treatment of nervous system disorders are psychobiotics that modulate the intestinal microbiome and positively impact the functioning of the microbiota-gut–brain axis [34]. There is ample evidence of their beneficial role in patients with mental disorders. An example is increasing the neurotransmitter concentration in the intestine, which can decrease the concentration of tryptophan in the plasma and stimulate the cells of the intestinal lining to release metabolites, thus improving the emotional well-being of patients [24,47]. SCFAs are likely to act as epigenetic modulators through histone deacetylases [48]. The third system of psychobiotics interaction is known as the hypothalamic–pituitary–adrenal axis (HPA). This likely plays a crucial role in the onset of mood disorders and cognitive problems. The gastrointestinal microbiota are a critical factor in HPA axis regulation to reduce the concentration of the stress hormone cortisol [49,50]. In the case of HPA axis dysfunction, the production and function of stress-related hormones are disrupted. The molecular basis for the role of the gut microbiota in maintaining mental health and the influence of psychobiotics on selected disease entities are presented later in the article.

The growing interest in psychobiotics and mental health has raised many questions for science, most of which remain unanswered. This review aims to collect information on the influence of psychobiotics on the gut–brain axis in selected disease entities (depression, Alzheimer’s disease, Parkinson’s disease, and autism spectrum disorders).

## 2. Materials and Methods

### Search Strategy

The search for articles was conducted from June 2021 to April 2022 and the results were structured as proposed by the Preferred Reporting Items for Systematic Reviews and Meta-Analyses (PRISMA) 2020 [51]. The articles included in the study were searched for using three electronic databases: Pubmed, Scopus, and Science Direct. Due to the variety of disease entities, the search for articles involved many phrases. The following combinations of the disease entities “depression” or “depressive” or “autism” or “Parkinson” or “Alzheimer” with words such as “probiotics” and “psychobiotics” were searched. The databases were searched independently by two researchers (NWK and AB) and cross-checked. Preliminary inclusion criteria were met if the selected disease entity appeared in the article title or abstract Articles that were duplicated in the searched databases were manually removed. The following data were extracted: book chapters, review articles, mini-reviews, encyclopedia, conference abstracts, correspondence, short communications, discussion, editorials, letters, notes, and short surveys. Only studies published in English were selected. The abstracts of the remaining publications were then screened.

The analysis included papers in which the study participants had a clear prior diagnosis of any of the mentioned conditions and the primary outcome studied was a change in cognitive function. The exclusion criteria were: animal studies and the lack of association between the disease and probiotics or psychobiotics. No publication year restriction was applied. Additionally, studies showing the correlation between the disease entity and probiotics, but not the direct impact of bacteria on the nervous disease, were excluded from the review, e.g., probiotics affected only coexisting symptoms of depression from the digestive system (e.g., constipation). 

Studies were graded to assess quality using a previously validated instrument, the Downs and Black checklist for quality of studies (Quality Index, QI) [52].

## 3. Results and Discussion 

**Figure 3:**
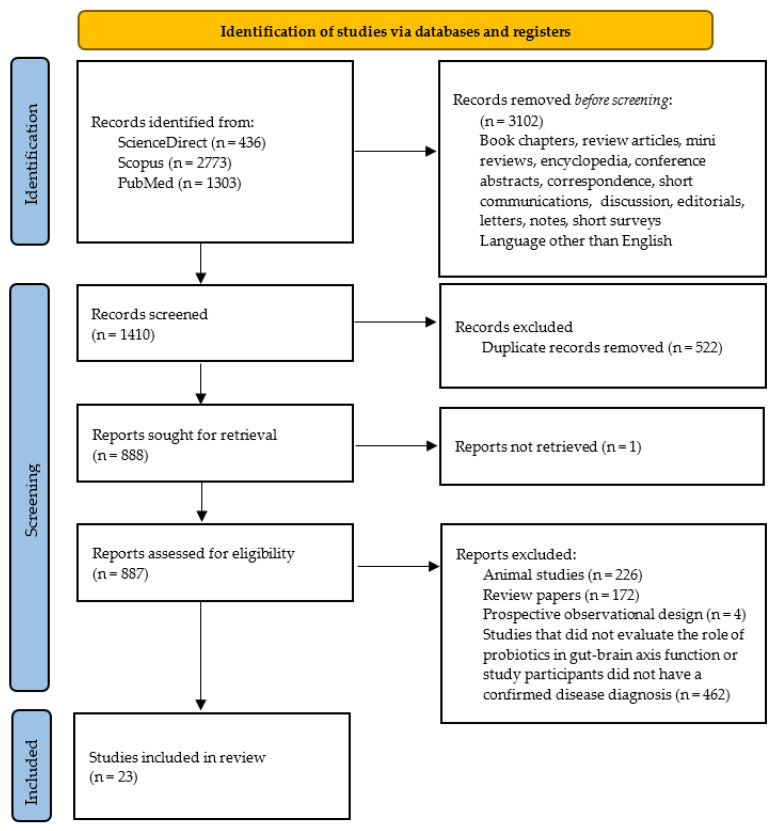
PRISMA flow diagram for systematic review depicting the phases of the identification of studies.

Among the 4512 citations obtained, 3102 did not meet the inclusion criteria and were excluded before the screening. Of the remaining 1410 articles, 522 were removed because they were duplicates in the databases searched. One paper was excluded because the full text was not available. After checking the titles and abstracts, 864 publications were excluded because they involved animal studies (n = 226), were review papers (n = 172), or were prospective observational designs. Studies in which the participants did not have a diagnosis of depression, Parkinson’s disease, Alzheimer’s disease, or autism, or which did not aim to assess the effect of probiotics on the severity of disease symptoms (n = 462), were also not included in this review. Twenty-three studies met the inclusion criteria. Figure 3 shows the results of the selection.

The mean (SD) quality index scores for randomized and non-randomized trials were 22.8 and 14.6, respectively. The mean scores and range of scores for each subscale are shown in Table 2. The quality index score for all manuscripts is presented in Supplementary Materials Table S1.

Table 3 presents the characteristics of the studies selected for systematic review. The studies were divided according to the disease unit, and their results were analyzed separately.

### 3.1. Depression

We reviewed 526 publications found in journal databases for the use of probiotics in people with depression. Sixteen studies met the inclusion criteria (Table 3), of which nine were randomized placebo-controlled trials (one unblind, eight double-blind, and one triple-blind), five were open trials, and one was a pilot study. The study included 909 people, with the group size ranging from 10 to 119 participants. The persons participating in the studies had identified anxiety and depressive symptoms, or were diagnosed with depression of varying severity. Participants were screened according to Diagnostic and Statistical Manual of Mental Disorders (DSM) criteria, using the Beck Depression Inventory (BDI), Outcome Questionnaire 45 (OQ45), Quality of Life (QoL), Mini International Neuropsychiatric Interview (MINI), Hamilton Depression Rating Scale-24 Items (HAM-D), or Hospital Anxiety and Depression Scale (HADS). In two publications, the study group consisted of patients with treatment-resistant major depressive disorder [53,54]. In four studies, additional inclusion criteria were patients diagnosed with mild to moderate irritable bowel syndrome [55,56], schizophrenia [57], or patients with chronic gastrointestinal symptoms [58]. In five studies, the exclusion criterion was the use of antidepressants [55,58,59]. In six studies, participants took antidepressants during the intervention [53,54,60,61,62,63]. In the remaining cases, the study group was diversified [56], or there was no information about the number of people who were/were not undergoing pharmacotherapy for depression. The probiotics administered in six studies were single-strain [54,55,56,57,64,65], or contained two [60,61,62] or three [47,53,59] strains of bacteria in six, three, and two studies, respectively. In the remaining studies, the multi-strain preparation consisted of more than seven strains of bacteria. The most frequently used were strains belonging to the genus *Lactobacillus* or *Bifidobacterium*. The participants of six studies were asked not to consume other supplements during the intervention [47,55,57,60,61,62].

The intervention lasted from 4 weeks to 90 days, with the 8-week period being the most frequent in the studies (10 out of 15). Changes in psychometric symptoms from the baseline were assessed using the BDI [43,47,53,54,60,61,62,66], OQ45 [53], QoL [53], Hamilton Rating Scale for Depression (HAM-D) [54,55,56,63,64,65,66], Montgomery–Asberg Depression Rating Scale (MADRS) [55], 11-item Centre for Epidemiological Studies–Depression Scale (CES-D) [55], Brief Psychiatric Rating Scale (BPRS) [56], Beck Anxiety Inventory (BAI) [43,54], HADS and Positive and Negative Syndrome Scale (PANSS) [57], MINI, Depression Anxiety Stress Scale 21 Items (DASS-21) and Leiden Index of Depression Sensitivity-Revised (LEIDS-R) [43], Symptom Checklist (SCL-90) [64,66], Perceived Stress Scale (PSS-10) [64], HADS [59], Montgomery-Åsberg Depression Rating Scale (MADRS) and Quick Inventory of Depressive Symptomatology (QIDS-SR16) [59], or the Depression and Somatic Symptoms Scale (DSSS) [65].

Probiotic supplementation improved the depressive symptoms in all studies, except for one [64], where no significant changes were found in patients diagnosed with major depressive disorders (based on the HAM-D, SCL-90, and PSS-10 scores). There was a relevant difference between the treatment and placebo groups in five of the nine placebo-controlled trials [47,55,56,60,61,62]. However, in the case of two randomized, triple/double-blind, controlled placebo trials and one randomized, unblind placebo-controlled study, no significant differences were observed between the compared groups in the assessed depression changes by the BDI, BAI, DASS stores [43], MINI [43,66], and HAM-D [63]. This indicates a non-specific positive effect of the probiotic in reducing the symptoms of depression, not different from the effect of a placebo.

The results of tests assessing the psychobiotic effect of probiotic bacteria, carried out before the beginning and at the end of the supplementation period, showed statistically significant decreases in the score, indicating a positive treatment effect [53,54,58,65]. In a study with the impact of probiotics measured in the middle and at the end of the intervention period, statistically significant changes in the HADS scale were observed only in the first period of treatment [57]. A statistically significant improvement was observed in the PANSS score, regardless of the evaluation period. A study in patients with moderate clinical depression who did not respond to treatment showed a statistically significant reduction in the MADRS and QIDS-SR16 scores after 4 weeks. However, the reduction was no longer significant after another 4 weeks [59].

Nine studies assessed side effects, but in one [66], the authors did not provide information on their possible occurrence/absence. No studies found severe/serious side effects of taking probiotics. Two studies reported no side effects. In one study, 2 out of 45 patients experienced mild and transient side effects [54], similar to another study (burning sensation in throat), where symptoms were rare (<5%) [58]. In two studies [61,62], side effects were reported by 9 out of 28 patients using probiotics (no such side effects were reported in the placebo group). In another two studies, the side effects were not statistically significantly more frequent in the treatment group than in the placebo group [43,64]. The exception was the symptom of experienced somnolence, which occurred statistically more frequently in participants taking probiotics [43].

Depression is a global disease that affects more women than men. According to estimates, approximately 5.0% of adults suffer from depression (5.7% among adults older than 60 years) [67]. The standard treatment of depressed patients is based on Selective Serotonin Reuptake Inhibitors (SSRIs) [68]. However, such therapy is not always sufficient. Scientists have been searching for new therapeutic solutions for patients with depression. Recent years have brought interest in and the development of work on the microbiota–gut–brain axis, especially the changes in the gut microbiome composition in patients with depression [69,70]. People with depression most often have a reduced number of bacteria of the genera *Bifidobacterium* and *Lactobacillus* [53,71,72]. There has been interest in using probiotics as antidepressants [73]. In 2013, Dinan et al. [74] introduced the concept of a psychobiotic, defined as a classic probiotic yielding neurobehavioral- or psychiatry-beneficial functions. The exact interactions of the gut microbiome in depressed patients are still unknown. However, scientists have drawn attention to the use of psychobiotics in the clinical treatment of depression [73].

Two studies did not report any adverse effects from the psychobiotic supplementation [75,76]. In other studies, isolated cases of mild adverse events were reported. The first studies assessing the effect of probiotics on the alleviation of depression were presented in a mouse model [77,78,79,80] and a rat model [81,82,83,84], using one bacterial strain [79,81]. Animal model studies have produced promising results. For example, Ding et al. [78] showed that *Akkermansia muciniphila* supplementation alleviated the symptoms of depression in mice through a mechanism related to the regulation of metabolites and the gut microbiota. In turn, Birmann et al. [80] showed that *Komagataella pastoris* KM71H supplementation in mice prevented depression-like behavior. Gu et al. [84] obtained similar results in a rat model. Researchers have shown that supplementation with *Lactobacillus casei* may protect against depression in rats, which is likely related to changes in the gut microbiota composition [84].

The next stage of the research was clinical trials involving patients diagnosed with depression. Some studies only evaluated the effect of probiotics on gastrointestinal symptoms, such as constipation, without checking the impact on depressive behavior. For example, a study by Zhang et al. [85] focused on assessing the effect of *Lacticaseibacillus paracasei* strain Shirota on constipation in depressed patients. The researchers showed that the 9-week daily supplementation positively affected the symptoms of constipation faced by patients with depression [85]. The above analysis aimed to evaluate the impact of probiotics and psychobiotics on depressive behavior. Most clinical trials have many limitations, which the authors also emphasize. One of the main limitations is often the small study group. Chen et al. [65], despite conducting studies on a small number of patients, confirmed the positive role of *Lactobacillus plantarum* PS128 supplementation in patients with depression. On the other hand, Romijn et al. [86] reported no significant changes after supplementation with *Lactobacillus helveticus* and *Bifidobacterium longum*, both in depression (depression not diagnosed by a doctor) and in the level of inflammatory markers. Another aspect that makes it difficult to compare studies on psychobiotics are differences in methodology and the characteristics of the research participants. The works included in our analysis were interventions conducted for four weeks to 90 days, with the period of 8 weeks being the most frequently recorded (10 out of 15). Such a discrepancy in the period of supplementation does not allow specifying the appropriate period of psychobiotic therapy as “antidepressants”. It also does not allow the unification of the results obtained by researchers and the possibility of subjective comparison. Changes in psychometric symptoms from the baseline were assessed using the BDI [43,47,53,54,60,61,62,66], OQ45 [53], QoL [53], Hamilton Rating Scale for Depression (HAM-D) [54,55,56,63,64,65,66], Montgomery–Asberg Depression Rating Scale (MADRS) [55], 11-item Centre for Epidemiological Studies–Depression Scale (CES-D) [55], Brief Psychiatric Rating Scale (BPRS) [56], etc. Another comparative difficulty in the presented studies is the diversity of the probiotic strains used (Table 3). Each strain possesses different properties, so the effects of its supplementation among patients with depression may vary. A relevant factor is also the number of strains in such a preparation [54,55,56,57,64,65]. The use of multiple probiotic strains showed a better effect than the use of a single one [60,61,62].

Probiotic-mediated reduction in depression symptoms is also associated with age. A metanalysis by Zhu et al. [73] found that probiotics showed noticeable antidepressant-like effects only in participants under the age of 60. None of the studies presented by us dealt with differences between the sexes.

Despite the availability of a large amount of data, the exact mechanism of action of probiotics in alleviating depression symptoms still needs to be confirmed. The aspect to consider is the severity of symptoms and the effects of psychobiotics, as well as dosages. In the works presented by us, this aspect was not elaborated on. Chahwan et al. [43] divided the study group into patients with mild and moderate depression. The researchers [43] found no significant differences between groups in the measures of depression, as well as taxonomic diversity in the gut microbiome.

A significant aspect of the work by Chen et al. [65] was the evaluation of the alteration of biomarkers of gut permeability and gut microbiota. The knowledge of the gut microbiota composition prior to and after the probiotic supplementation provides important information about the gut microbiome and its influence on depressive disorders. Otaka et al. [87] used *Lacticaseibacillus paracasei* strain Shirota. The researchers found the link between the reduction in depressive symptoms and the gut microbiome. Reduced symptoms of depression were partially related to the increase in Actinobacteria in the gut microbiome [87]. Most of the studies illustrated by us showed a positive effect on depressive behavior among patients. Only Rudzki et al. [64] showed no significant improvement in patients with depression receiving supplementation with *Lactobacillus plantarum* 299v. However, this research is only the foundation of this rapidly evolving field, where further, well-chosen research is essential. Future directions should include larger study groups, the division of results by gender (due to the differences in the gut microbiome composition), and the side-effects of long-term supplementation. In our opinion, supplementation with probiotic strains can complement the standard therapy of patients with depression.

### 3.2. Alzheimer’s Disease

Out of 146 publications on Alzheimer’s disease and probiotics, four studies met the inclusion criteria (Table 3), three of which were randomized, double-blind, controlled trials conducted in Iran [88,89,90], and one of which was an uncontrolled clinical investigation conducted in Brazil [91]. In total, the analysis covered the results obtained for 200 people diagnosed with Alzheimer’s disease according to the criteria of the National Institute on Aging–Alzheimer’s Association [92]. Three studies [88,89,90] also included the NINCDS-ADRDA criteria [93]. The sample sizes in the randomized trials ranged from 25 to 30 in the probiotic supplement group, and from 23 to 30 in the placebo control group. In the case of the uncontrolled clinical investigation, the trial that completed the study was small—13 people. In each of the studies, the researchers assessed the influence of a mixture of probiotics, differing in both their qualitative and quantitative composition. In one study, the participants received probiotics in combination with selenium [89]. The duration of the intervention was similar and ranged from 84 to 90 days. Three studies [88,89,91], compared cognitive functions using the Mini-Mental State Examination (MMSE) test before and after probiotic supplementation. One study additionally evaluated [91] immediate and delayed memory, visual abilities and spatial, abstract, and visual–constructional functions, as well as executive and linguistic functions. In the fourth paper [90], Test Your Memory (TYM), introduced by Brown et al. [94], was used to determine the level of cognitive abilities and detect AD.

In addition, all studies also analyzed biomarkers of oxidative stress and inflammation—one study [88] assessed metabolic profiles and another [91] estimated molecular and cell integrity. Any study analyzed fecal microbiota before and after probiotic supplementation.

Two clinical trials [88,89] observed a significant improvement in the MMSE result in the group of people taking probiotics compared with the control group. In contrast, in the study evaluating the results of TYM, probiotic supplementation only slightly improved the cognitive indicators [90]. A beneficial effect on all examined cognitive functions was documented by Ton et al. [91]. The other test results were adequate for the results concerning cognitive functions—supplementation, which had no significant impact on the TYM results, did not affect the biochemical factors tested [90]. Three studies [88,89,91] showed that the use of probiotics in AD patients had a positive effect on some biomarkers of oxidative stress and inflammation, or on lipid profiles, as well as the induction of DNA repair and reduction in apoptosis.

Alzheimer’s disease is the most common chronic neurodegenerative disorder and accounts for approximately 60–70% of all cases of dementia [95]. The neuropathological hallmarks of the disease are extracellular amyloid plaques in the brain containing amyloid-β and neurofibrillary tangles containing the tau protein. Following the aggregation of amyloid plaques and neurofibrillary tangles in the brain, there is an increase in local microglia activation, leading to inflammation [96]. Previous studies indicated that peripheral infections or oxidative stress, which is conducive to the further production of myloid-β, may accelerate neurodegeneration in Alzheimer’s disease [97,98,99]. Moreover, researchers have observed a reduction in the number and diversity of bacteria in the intestinal tract in Alzheimer’s disease. The reduced number of intestinal bacteria leads to a decrease in the content of intestinal hormones in the plasma, e.g., ghrelin, which prevents neurodegeneration, or leptin and glucose-dependent insulinotropic polypeptide, with neuroprotective effects [100]. The gut microbiome of Alzheimer’s patients contains a reduced number of butyrate-synthesis bacteria (*Butyrivibrio hungatei* and *B. proteoclasticus*, *Eubacterium eligens*, *E. hallii* and *E. rectale*, *Clostridium* spp. strain SY8519, *Roseburia hominis*, and *F. prausnitzii*) and also higher numbers of taxa that contribute to pro-inflammatory conditions (including *Odoribacter splanchnicus* and *Bacteroides vulgatus*). Such a composition of the gut microbiome leads to brain inflammation and progressive cognitive decline [101]. Studies on animal models show that the gut microbiota, by producing phenolic acids and fiber metabolism to short-chain fatty acids, can inhibit disease progression. For example, 3-hydroxybenzoic acid and 3-(3’-hydroxyphenyl) propionic acid disrupt the misfolding of β-amyloid peptides that form protein aggregates. Valeric acid, butyric acid, and propionic acid effectively disrupt the interactions between β-amyloids, so peptides cannot aggregate into neurotoxic proteins [102]. Therefore, scientists have suggested that probiotics may act on the CNS via the gut–brain axis. Their intake could positively affect brain activity by the synthesis of neurotransmitters, expression of receptors producing neuromodulators and neurotransmitters, anti-inflammatory, and antioxidant effects, or the impact on the endocrine system. Studies in animal models of AD have shown that probiotics can alleviate memory and cognitive deficits [103,104,105,106]. The number of publications on the influence of probiotic supplementation on mental health improvement in people with Alzheimer’s disease is limited. This systematic review shows the positive impact on cognitive functions only in patients with mild Alzheimer’s disease [88,89]. Probiotics did not improve cognitive functions, inflammatory and anti-inflammatory parameters, or oxidative and antioxidant biomarkers in the study group of patients with severe (83.5%) and moderate forms of the disease [90]. The varied results may be due to the different tests performed in the cognitive assessment. The greater sensitivity of the TYM test than that of the MMSE in the dementia detection could affect obtaining results negating the role of probiotics in the alleviation of AD symptoms. A significant drawback of one study was the lack of a control group, which made it difficult to confirm the effectiveness of improving cognitive functions in patients. The doses of probiotics used may have also influenced the observed differences between studies. One study assessed the synergistic effect of probiotics and selenium, but results regarding supplementation with probiotics alone were missing, which further complicated the analysis. Moreover, the administered supplements differed in their composition (different strains of microorganisms, different number of strains in the preparation), as well as the method of administration. However, the World Gastroenterology Organization does not provide the required dosage and frequency of probiotic supplementation [107] that have a beneficial effect on human health. Therefore, it is difficult to determine what dose in this type of intervention would be effective. In addition, many factors, such as the type or carrier, may affect the role of probiotics. In the analyzed publications, there was no information regarding the control of fermented food consumption by the participants, which could also impact the obtained results. Additionally, the negative effect of taking probiotics, which may occur in the elderly, has not been assessed.

### 3.3. Parkinson’s Disease

Among 61 studies evaluating the importance of probiotic use in Parkinson’s disease, one study by Lu et al. [108] met the inclusion criteria (open-label, single-arm, baseline-controlled trial) (Table 3). The study included 25 patients with Parkinson’s disease (mean age 61.84 ± 5.74 years) diagnosed using the Unified Parkinson’s Disease Rating Scale (UPDRS), modified Hoehn and Yahr scale, and change in patient “ON–OFF” diary recording. The study included a psychobiotic *Lactobacillus plantarum* PS128 at a dose of 30 billion colony-forming units twice per day for 12 weeks. After the completion of the supplementation, the parameters of the patients included in the study were assessed, i.e., the Unified Parkinson’s Disease Rating Scale UPDRS part III, motor scores, changes in patient’s “ON–OFF” diary recordings, and modified Hoehn and Yahr scale (mHYS). Additionally, the biochemical parameters of the patients, including the level of C-reactive protein, the plasma myeloperoxidase (MPO), and urinary 8-hydroxy-2‘deoxyguanosine, were checked. After 12 weeks of psychobiotic supplementation, the results of patients with Parkinson’s disease improved. Seventeen patients exhibited improvement in the motor (UPDRS parameter) and non-motor results. The results of the biochemical tests were also satisfactory. *L. plantarum* PS128 supplementation reduced the plasma myeloperoxidase and urine creatinine levels. The study did not include the analysis of fecal microbiota before and after supplementation with a psychobiotic [108].

Parkinson’s disease is the second-most-common neuropsychiatric disorder [109]. The neuropsychiatric symptoms include depression, anxiety, apathy, cognitive decline, dementia, and psychosis. Gastrointestinal complaints are frequent symptoms that accompany the disease [110]. One of the mechanisms of neuronal death in Parkinson’s disease is based on oxidative stress [109]. In patients with Parkinson’s disease, the gut microbiota change. There is a reduction in the number of bacteria of the genus *Prevotellaceae* that participate, among others, in the synthesis of vitamin B1, which is responsible for the release of dopamine. The lower number of the bacteria is associated with a decrease in ghrelin secretion in the early stages of the disease. This hormone is primarily responsible for, among other things, supporting the proper functioning of dopaminergic cells. Patients have an increased number of bacteria belonging to the genus *Bifidobacterium* [111,112] and the family *Verrucomicrobiaceae*, including the genus *Akkermansia*, which decompose mucin responsible for moisturizing the intestinal wall and creating a barrier between epithelial cells and the surface of the lumen. 

The study by Lu et al. [108] was the first study in humans with confirmed Parkinson’s disease to consider the effects on motor functions. Despite the small number of patients, the study reported a positive influence of psychobiotic supplementation on motor functions [94]. There are studies that have only investigated the effects of probiotics on the gastrointestinal function of Parkinson’s patients and studies based on an animal model.

Probiotic supplementation alleviated gastrointestinal symptoms (abdominal pain, constipation, or flatulence) that are common among Parkinson’s patients [113,114]. The study by Cassani et al. [113] showed that supplementation with *Lactobacillus casei* Shirota reduced abdominal pain and normalized the table and bowel movements in patients with Parkinson’s disease. In turn, Ghyselinck et al. [115] assessed the fecal microbiome of patients with Parkinson’s disease 48 h after supplementation with a four-strain probiotic suspension. Researchers reported higher levels of Bacteroidetes and lower levels of Firmicutes among Parkinson’s disease patients compared with healthy controls [115]. Tan et al. [116], in a randomized controlled trial, showed that a multi-strain probiotic was effective in treating constipation in Parkinson’s patients. Similar results were documented by Ibrahim et al. [117]. Studies on an animal model (mouse or rat) are more abundant [118,119,120,121,122]. The study by Liu et al. [123] demonstrated a positive effect of a polymannuronic acid prebiotic plus *Lacticaseibacillus rhamnosus* GG during a five-week supplementation in a mouse model of Parkinson’s disease. The synbiotic proposed by the researchers showed a neuroprotective effect (protection against the loss of dopaminergic neurons) [12]. Wang et al. [124] observed that *Lactobacillus plantarum* DP189 improved behavioral abilities and increased the levels of 5-hydroxytryptamine and dopamine in mice with Parkinson’s disease. Ma et al. [125], in a rat model, found that the psychobiotic *Lactiplantibacillus plantarum* PS128 played a crucial role in dopaminergic neuroprotection and increased the availability of neurotransmitters. Research in animal models shows promising results (improvement in motor functions in the Parkinson’s disease model), which is a reason for further studies in humans.

Since the dysbiosis of the intestinal microbiota affects the progression of Parkinson’s disease, supplementation or supportive treatment with probiotic preparations may have a beneficial impact on the functioning of patients. The above studies indicate that probiotic supplementation helps to maintain the intestinal microbiome balance and reduce constipation or abdominal pain. According to us, probiotic supplementation in people with diagnosed Parkinson’s disease is a good practice for supporting treatment.

### 3.4. Autism Spectrum Disorder

We identified 186 papers on the use of probiotics in the treatment of autistic spectrum disorder. Based on the review of titles and abstracts, five publications met the inclusion criteria: three randomized, double-blind, controlled placebo trials, one real-world experience, and one prospective, open-label study (Table 3). A total of 330 individuals with a diagnosis of ASD were confirmed by the Diagnostic and Statistical Manual of Mental Disorder (DSM-IV/V) [75], Autism Diagnostic Observation Schedule, Second Edition (ADOS-2) [75,126,127,128], and the Autism Diagnostic Interview–Revised (ADI-R) [75,76,126]. The people included in the study were under 25 years of age. In one randomized, controlled placebo trial, the participants were classified into groups with (GI) or without (NGI) gastrointestinal symptoms [127]. The duration of the intervention ranged from 4 weeks to 6 months. In one study, the inclusion criterion was probiotic supplementation for at least three months [127]. In two studies, the exclusion criteria included taking probiotics in the last 2 or 4 weeks [75,76]. In one of the studies mentioned, the participant was also not allowed to eat yogurt or take antibiotics two weeks before starting the study, and throughout the entire study [76]. The use of an antibiotic during the intervention resulted in the withdrawal of one patient from the study [75]. Other studies did not indicate the above-mentioned exclusion criteria. More, in one study, 48.4% of the participants taking the probiotics underwent antibiotic therapy [127]. The probiotic species reported in all studies except for one [128] was *Lactobacillus plantarum*. In three cases, it was the only ingredient [75,76,126], while the remaining participants received multi-strain preparations. In one study, 26 of 131 patients received probiotics, the qualitative and quantitative composition of which was not given [126]. In addition, one study started using a probiotic with oxytocin after 16 weeks to compare the effects of the probiotic alone and the combination therapy [75].

The primary outcomes of the studies were the disease severity as measured by the Clinical Global Impression (CGI) [75,76,126], the Child Behavior Checklist (CBCL) questionnaire [76], and the Autism Treatment Evaluation Checklist (ATEC) [128]. Two randomized, double-blind, controlled placebo trials exhibited improvements in the ABC and SRS, ADOS-CSS, and CGI scores [75,127], with a change in the ABC and SRS scores only for the group using the combination therapy [75]. In these studies, no statistically significant differences in the severity of autism were observed between the groups receiving the probiotic and the placebo. The exception was the comparison of symptoms measured on the ADOS-CSS scale for the GI group and the placebo group [127]. One randomized, controlled placebo trial reported no changes in disease severity (assessed by CGI questionnaires). Changes were noted in the SRS results and several items of the ABC, SRS, CBCL, and SNAP-IV questionnaires [76]. The other two studies showed a clinically significant improvement in the CGI and ATEC scores, indicating the importance of supplementation in autism spectrum disorders [126,128]. In addition, two studies found a correlation between the participant’s younger age and the positive effect of a probiotic [76,126].

Autism Spectrum Disorder (ASD) is a serious neurodevelopmental disorder characterized by impaired communication and social interaction, limited and repetitive patterns of behavior, interests, and activities. The disease is often accompanied by digestive problems, such as diarrhea, constipation, flatulence, or autistic enteritis [129]. Untreated GI symptoms exacerbate behavioral problems in children [130]. Currently, there are no recognized effective methods of treating the disorder [131].

In each study, the probiotic intervention improved the results in the scales assessing the severity of autism. Beneficial effects included increased attention, communication skills, sociability, interaction and personal autonomy of the patient, sensory/cognitive awareness, anxiety, rule-breaking behavior, hyperactivity/impulsivity, and opposition/aversion. The methods used in the assessment differed between studies. Some were based on interviews or questionnaires, which, in the case of national minorities and the existing linguistic and cultural barriers, could affect the results [75]. Despite the positive effect of probiotics on the alleviation of ASD symptoms, in the studies involving control groups, the results did not differ significantly from those obtained for the placebo groups. To analyze the impact of supplementation, it is also necessary to randomize study participants, which was missing in two studies [126,128].

The age of the participants in individual studies showed high heterogeneity [75]. Therefore, it is difficult to determine the effectiveness of treatment and compare the results obtained for a group of preschoolers [127] and school-age children or adolescents [76]. 

A positive aspect of the obtained results was the lack of side effects or their occurrence in a mild and transient form. However, none of the studies investigated the influence of supplementation suspension on the maintenance of improvement or worsening of ASD symptoms. Most publications did not mention the exclusion of functional foods or supplements during the course of the intervention that could have had an additional effect on the study’s result. Only in one study were participants required to eliminate yogurt from their diet [76]. Therefore, the use of probiotics as a therapy supporting the treatment of people with autism spectrum disorders merits further investigation (taking into account the limitations of the research conducted so far). A sufficiently large test group is also essential to obtain reliable results. The assessment of the gut microbiota composition of the study participants, which may correlate with the severity of autism symptoms, was included only in two analyzed studies [75,128].

**Table 3:** A collection of clinical studies on the effects of probiotic supplementation in people diagnosed with selected disease entities.

	Type of Examination	Study Population	Preparation/Probiotic Bacteria	Duration of the Intervention	Results	Statistical Significance	References
**DEPRESSION**	Pilot study	12 patients diagnosed with SSRI-treatment-resistant depression (mean age, 19.8 ± 5.7 years)	Magnesium orotate (1600 mg), and probiotics (*Lactobacillus acidophilus*, *Bifidobacterium bifidum*, amd *Streptoccocus thermophiles*) (total CFU 2 × 10^10^ divided between 2 daily doses)	16 weeks (active intervention administered for 8 weeks)	Reduction in depressive symptoms Improved quality of life after the end of the intervention	SSD in 2 scores after 8 weeks of supplementation (both *p* = 0.005) SID in BDI score in relapse after 16-week follow-up (*p* = 0.068)	[53]
	Double-blind, placebo controlled, randomized, multi-centre, pilot clinical study	40 patients with mild to moderate IBS and MDD (mean age, 40.36 ± 10.28 years)	*Bacillus coagulans* MTCC 5856 (2 × 10^9^ CFU) (1 tablet per day)	90 days	Improvement in IBS and depression symptoms	SSD in 3 scores in the treated group (each *p* ≤ 0.001)	[55]
	Placebo-controlled, double-blind randomized controlled trial	45 patients with mild to moderate IBS and MDD (mean age, 51.32 ± 16.11 years)	*Bifidobacterium breve* CCFM1025 (total CFU 10^10^) (1 sachet per day)	4 weeks	Better antidepressant-like effect Reduced gastrointestinal symptoms	SSD in 3 scores in the treated group (each *p* < 0.001) and in 2 scores in the placebo group (*p* < 0.001; *p* = 0.036)	[56]
	Randomized, double-blind, controlled placebo trial	40 patients with a diagnosis of major depressive disorder (age range: 20–55)	*Lactobacillus acidophilus* (2 × 10^9^ CFU/g), *Lactobacillus casei* (2 × 10^9^ CFU/g), and *Bifidobacterium bifidum* (2 × 10^9^ CFU/g) (1 capsule per day)	8 weeks	Reduction in the Beck Depression Rating Scale Improvement in insulin function Reduction in oxidative stress	SSD in BDI score in the treated group (*p* = 0.001)	[47]
	Prospective open-label trial	40 patients with treatment-resistant major depressive disorder (mean age, 44.2 ± 15.6 years)	*Clostridium butyricum* MIYAIRI 588 (CBM588) (20 mg orally/twice a day for the first week; 20 mg orally/three times a day from weeks 2 to 8)	8 weeks	Significant improvement in depression (greater treatment efficacy in patients with a lack of response to previous antidepressants)	SSD in BDI score in the study group (*p* < 0.001)	[54]
	Open-label single-arm study	29 outpatients with schizophrenia with anxiety and depressive symptoms (mean age, 45 (16) years)	*Bifidobacterium breve* A-1 (5.0 × 10^10^ CFU) (2 sachets per day)	8 weeks (active intervention administered for 4 weeks)	Potential effect in improving anxiety and depressive symptoms	SSD in 2 scores at 4 weeks (*p* = 0.037; *p* = 0.004) and in 1 score during the post-observation period (*p* = 0.004)	[57]
	Three-arm parallel design, placebo-controlled, double-blind randomized controlled trial	81 patients with mild to moderate major depression (mean age, 36.5 ± 8.03 years)	*Lactobacillus helveticus* R0052 and *Bifidobacterium longum* R0175 (≥10 × 10^9^ CFU) (1 sachet per day)	8 weeks	Improved depression symptoms Decreased serum kynurenine/tryptophan ratio	SSD in BDI score in the treated group (*p* = 0.008)	[61]
	Double-blind, placebo-controlled, single-center, parallel design randomized controlled trial	110 patients with a diagnosis of major depression (mean age, 36.15 ± 7.85 years)	*Lactobacillus helveticus* R0052 and *Bifidobacterium longum* R0175 (≥10 × 10^9^ CFU) (1 sachet per day)	8 weeks	Significant decrease in BDI score No effect on inflammatory marker levels Secrease in urinary cortisol levels	SSD in BDI score in the treated group (*p* = 0.04)	[60]
	Double-blind, randomized controlled trial	78 patients with low to moderate depression (mean age, 36.0 ± 9.0 years)	*Lactobacillus helveticus* R0052 and *Bifidobacterium longum* R0175 (≥10 × 10^9^ CFU) (1 sachet per day)	8 weeks	Improved depression symptoms	SSD in BDI score in the treated group (*p* = 0.012)	[62]
	Randomized, triple blind, controlled placebo trial	71 participants with mild to severe depression (mean age, 36.65 ± 11.75 years in probiotic group)	*Bifidobacterium bifidum* W23, *Bifidobacterium lactis* W51, *Bifidobacterium lactis* W52, *Lactobacillus acidophilus* W37, *Lactobacillus brevis* W63, *Lactobacillus casei* W56, *Lactobacillus salivactocarius* W56, *Lactobacillus casei* W56, *Lactococcus lactis* W19, and *Lactococcus lactis* W58 (total cell count 1 × 10^10^ CFU) (2 sachets per day)	2 months	Reducing symptoms of depression, anxiety, and stress Lowering the depression sensitivity marker Changing the classification of depression from clinical to subclinical in the research group No changes in the composition of the microbiota	SID in 3 scores in the treated group compared to the placebo group (each *p* > 0.05)	[43]
	Double-blind, randomized, placebo controlled trial	79 participants with major depressive disorder (mean age, 39.13 ± 9.96 years in probiotic group)	*Lactobacillus plantarum* 299v (1 × 10^9^ CFU) (2 capsules per day)	8 weeks	Improvement in cognitive performance Decreased concentration of kynurenic acid	SID in 3 scores in the study group (*p* = 0.797; *p* = 0.218; *p* = 0.369)	[64]
	Single-center uncontrolled trial	83 patients with symptoms suggesting anxiety/depression (mean age, 43.9 ± 12.3 years)	*Bifidobacterium bifidum W23*, *Bifidobacterium lactis W52*, *Lactobacillus acidophilus W37*, *Lactobacillus brevis W63*, *Lactobacillus casei W56*, *Lactobacillus salivarius W24*, *Lactococcus lactis W19*, and *Lactococcus lactis W58* (over 2.5 × 10^9^ CFU/g) (1 sachet per day)	8 weeks	Anxiety and depression symptoms significantly improved	SID in tested score in the study group (*p* < 0.001)	[58]
	Open-label exploratory study	10 participants in a current episode of MDD (mean age, 25.2 ± 7.0 years)	*Lactobacillus helveticus* *R0052* (90%) and *Bifidobacterium longum R0175* (10%) (3 × 10^9^ CFU) (1 sachet per day)	8 weeks	Improved overall mood and anhedonia Reduced anxiety, and improved sleep quality	SSD in 2 scores at 4 weeks (both *p* < 0.001) and SID at 8 weeks (*p* = 0.377; *p* = 0.126)	[59]
	Randomized placebo-controlled study	119 participants with a mild or moderate depressive episode (mean age, 32.9 ± 6.1 years)	*Lactobacillus casei* PXN 37, *Lactobacillus plantarum* PXN 47, *Lactobacillus rhamnosus* PXN 54, *Lactobacillus acidophilus* PXN 35, *Lactobacillus bulgaricus* PXN 39, *Lactobacillus helveticus* PXN 45, *Lactobacillus salivarius* PXN 57, *Lactobacillus fermentum* PXN 44, *Lactococcus lactis* ssp. *Lactis* PXN 63, *Streptococcus thermophilus* PXN 66, *Bifidobacterium bifidum* PXN 23, *Bifidobacterium breve* PXN 25, *Bifidobacterium longum* PXN 30, and *Bifidobacterium infantis* PXN 27 (2 × 10^9^ CFU) (3 capsules per day)	6 weeks	Reduction in depression symptoms Decrease in the levels of cortisol, dopamine, IL-6, TNF-α, and NO	SID in tested score in the main and the comparison group (*p* = 0.083)	[63]
	Open trial	11 patients with major depressive disorder (mean age, 39.4 ± 12.0 years)	*Lactobacillus plantarum* PS128 (3 × 10^10^ CFU) (2 capsules per day)	8 weeks	Depressive severity significantly ameliorated Markers of inflammation, gut permeability, and the composition of gutmicrobiota did not significantly change	SSD in 2 scores in the study group (*p* = 0.01; *p* < 0.001)	[65]
	Double-blind, randomized placebo-controlled trial	61 depressed patients (mean age, 43 ± 14.31 years)	*Bifidobacterium bifidum* W23, *Bifidobacterium lactis* W51, *Bifidobacterium lactis* W52, *Lactobacillus acidophilus* W22, *Lactobacillus casei* W56, *Lactobacillus paracasei* W20, *Lactobacillus plantarum* W62, *Lactobacillus salivarius* W24, and *Lactobacillus lactis* W19 (at least 7.5 × 10^12^ CFU), and 125 mg of D-Biotin (vitamin B7), 30 mg of common horsetail, 30 mg of fish collagen, and 30 mg of keratin (1 portion per day)	28 days	Probiotic therapy might help balance the microbiota composition in individuals with depressive disorders	SID in 3 scores in the treated group compared with the placebo group (*p* = 0.850; *p* = 0.660; *p* = 0.631)	[66]
**ALZHEIMER’S DISEASE**	Randomized, double-blind, placebo-controlled trial	60 patients (mean age, 77.67 ± 2.62 years in probiotic group)	Milk (200 mL per day) enriched with probiotic bacteria: *Lactobacillus acidophilus*, *Lactobacillus casei*, *Bifidobacterium bifidum*, and *Lactobacillus fermentum* (2 × 10^9^ CFU/g each)	12 weeks	Improvement in the mental status test Improvement in cognitive functions and selected metabolic indicators No improvement in indicators of oxidative stress and inflammation	SSD in MMSE score in the treated group compared with the placebo group (*p* < 0.001)	[88]
	Randomized, double-blind, placebo-controlled	79 patients (mean age, 76.2 ± 8.1 years in probiotic group)	Selenium (200 μg/day) and probiotic containing *Lactobacillus acidophilus*, *Bifidobacterium bifidum*, and *Bifidobacterium longum* (2 × 10^9^ CFU/day each)	12 weeks	Improvement in mental state Reduced levels of CRP protein, insulin, triglycerides, and LDL cholesterol Increase in antioxidant capacity	SSD in MMSE score in the treated group compared with the placebo group (*p* < 0.001)	[89]
	Randomized, double-blind, placebo-controlled trial	48 patients (mean age, 79.70 ± 1.72 years in probiotic group)	Two variants of the preparation: *Lactobacillus fermentum*, *Lactobacillus plantarum*, and *Bifidobacterium lactis*, or *Lactobacillus acidophilus*, *Bifidobacterium bifidum*, and *Bifidobacterium longum* (each with a total dosage of 3 × 10^9^ CFU) (1 of each capsule per day)	12 weeks	No improvement in cognitive performance or biochemical markers in patients with severe disease	SID in tested score in the treated and placebo group (*p* > 0.05)	[90]
	Uncontrolled clinical trial	13 patients with AD exhibiting cognitive deficit (mean age of women, 78.7 ± 3 years; mean age of men, 78 ± 7 years)	Probiotic-fermented milk: pasteurized milk inoculated with 4% kefir grains containing the species *Acetobacter aceti*, *Acetobacter* sp., *Lactobacillus delbrueckii delbrueckii*, *Lactobacillus fermentum*, *Lactobacillus fructivorans*, *Enterococcus faecium*, *Leuconostoc* spp., *Lactobacillus kefiranofaciens*, *Candida famata*, and *Candida krusei* (2 mL/kg/daily)	90 days	Improvement in memory, visual-spatial/abstraction abilities, and executive/language functions	SSD in MMSE score in the study group (*p* < 0.0001)	[91]
**PARKINSON’S DISEASE**	Open-label, single-arm, baseline-controlled trial	25 patients (mean age, 61.84 ± 5.74 years)	*Lactobacillus plantarum* PS128 (3 × 10^13^ CFU) (2 capsules per day)	12 weeks	Significantly improved UPDRS motor score and quality of life No obvious effect on non-motor symptoms	SSD in UPDRS scores in two parameters (*p* = 0.004; *p* = 0.012)	[108]
**AUTISM SPECTRUM DISORDER**	Real-world experience	131 autistic children and adolescents (age: 86.1 ± 41.1 months)	*Lactobacillus plantarum* PS128 (105 patients) (6 × 10^10^ CFU or 3 × 10^10^ CFU if patient’s weight was <30 kg) or other probiotics (not listed in the publication) (dose in the recommended range according to age, weight, and specific product)	6 months	Significant improvements in terms of global functioning of the patient Greater improvement in neurodevelopmental impairment scores in patients taking *Lactobacillus plantarum* PS128 than in those taking other probiotics	SSD in CGI severity in the study group (*p* < 0.001)	[126]
	Randomized, double-blind, controlled placebo pilot trial	35 individuals with ASD (mean age, 9.85 ± 4.91 years in probiotic group)	*Lactobacillus plantarum* PS128 (6 × 10^10^ CFU) (2 capsules per day) and oxytocin from 16 weeks	28 weeks	Reduced ASD core socio-behavioral symptoms and clinical global functioning Significant improvements in gut microbiome dysbiosis	SID in total scores measured by 2 scales in the study group (*p* = 0.077; *p* = 0.26) SSD in 1 score scale in the study group (*p* <0.05)	[75]
	Randomized, double-blind, controlled placebo trial	63 preschoolers with ASD (mean age, 4.16 ± 1.17 years in probiotic group)	“*Vivomixx*” (*Streptococcus thermophilus* DSM 24731, *Bifidobacterium breve* DSM 24732, *Bifidobacterium longum* DSM 24736, *Bifidobacterium infantis* DSM 24737, *Lactobacillus acidophilus* DSM 24735, *Lactobacillus plantarum* DSM 24730, *Lactobacillus paracasei* DSM 24733, and *Lactobacillus delbrueckii subsp. bulgaricus* DSM 24734) (4.5 × 10^11^ CFU) (2 packets or 1 packet/day in the first and in the following 5 months, accordingly)	6 months	No statistically significant changes in autism symptoms between probiotics and placebo group Significant modification of core ASD symptoms in group without gastrointestinal symptoms Alleviation of gastrointestinal symptoms, greater improvements in adaptive functioning, and sensory profiles than in the GI group	SID in tested score in the treated and placebo group (*p* = 0.16)	[127]
	Randomized, double-blind, placebo-controlled study	71 boys with ASD (mean age, 10.01 ± 2.32 years)	*Lactobacillus plantarum* PS128 (PS128) (3 × 10^10^ CFU/capsule)	4 weeks	Mitigation of some autism symptoms (hyperactivity/impulsivity, disruptive and rule-breaking behaviors)	SSD in 2 scores in the treated group (*p* = 0.04; *p* = 0.02) SID in other 2 scores in the treated group (*p* = 0.28; *p* = 0.1) and in 4 scores in the placebo group (*p* = 0.43; *p* = 0.2; *p* = 0.3; *p* = 0.86)	[76]
	Prospective, open-label study	30 autistic children (mean age, 84.77 ± 16.37 months)	*Bifidobacterium longum*, *Lactobacillus rhamnosus*, and *Lactobacillus acidophilus* (5 × 10^8^ CFU) (1 sachet per day)	3 months	Improvement in the severity of the ASD Positive impact on microbiota composition Reduction in the severity of gastrointestinal symptoms	SSD in tested score in the treated group (*p* = 0.0001)	[128]

BDI—Beck Depression Inventory; AD—Alzheimer’s disease; ASD—autism spectrum disorder; CFU—colony-forming units; CGI—Clinical Global Impression; CRP—C-reactive protein; GI—gastrointestinal symptoms; IBS—irritable bowel syndrome; LDL—low-density lipoprotein; MDD—major depressive disorder; MMSE—Mini-Mental State Examination; SID—statistically insignificant difference; SSD—statistically significant difference; SSRI—selective serotonin reuptake inhibitor; UPDRS—Unified Parkinson’s Disease Rating Scale.

## 4. Conclusions

For several years, the interest of the public in the impact of the intestinal microbiota on the health and functioning of the body has grown. The intestinal microbiome is a very extensive ecosystem that plays an essential role in maintaining homeostasis in the body [45]. Intestinal dysbiosis often accompanies various disease entities, especially modern ones, such as obesity, diabetes, and depression [4].

Psychobiotics are a special group of probiotics. They include strains of probiotic bacteria that affect the body through the gut–brain axis and are used in psychological disorders. They affect, among others, the tightness of the intestine, the synthesis of neurotransmitters and selected hormones, the stimulation of selected receptors, and some metabolic pathways [7].

Currently, drug administration is a more effective method of treating psychiatric diseases. The inclusion of psychobiotics in these conditions is promising. Such a solution has many positive effects, both for the patient (it improves their emotional well-being) and for the economy. Importantly, psychobiotics reduce the incidence of side effects.

Clinical trials showed that probiotic supplementation reduced anxiety, depression, and inflammation. Therefore, it is worth considering supporting pharmacotherapy with probiotic supplementation. Supplementation has improved cognitive functions, general mental state, and movement disorders in patients with Alzheimer’s disease and Parkinson’s disease. Furthermore, it has decreased the severity of gastrointestinal symptoms [71]. In autism spectrum disorders, probiotic preparation intervention improved behavior, reduced the severity of gastrointestinal symptoms, and the disease in general, and changed the microbiome’s composition. On the basis of the included studies, it cannot be stated whether the stronger the symptoms of a disease entity, the higher the effectiveness of psychobiotic preparations.

Collectively, probiotic supplementation has a positive impact on selected diseases. However, more research is needed to confirm its effectiveness. Supplementation with psychobiotics can complement standard pharmacotherapy.

## Figures and Tables

**Table 1:** General functions of neurotransmitters produced by the gut microbiota.

Gut Microbiota	Neurotransmitters Produced	Function	References
*Bifidobacterium infantis*, *Candida* spp., *Streptococcus* spp., *Escherichia* spp., *Enterococcus* spp.	Serotonin (5-HT; 5-hydroxytryptamine)	Involved in regulating behavioral and biological functions in the body, such as mood Plays a role in both psychological processes in the central nervous system (CNS) and peripheral tissues, such as the bone and gut	[24]
*Bacillus* spp., *Lactobacillus* spp.	Dopamine	Vital role in motor control, learning, memory formation, and the stress response Regulating carbohydrate and fat metabolism in the body Improvement in memory recovery	[25,26]
*Escherichia* spp., *Bacillus* spp., *Saccharomyces* spp.	Norepinephrine		
	Noradrenaline		
*Lactobacillus* spp., *Bifidobacterium* spp.	Gamma-aminobutyric acid (GABA)	Control of excitatory and inhibitory neurotransmission	[27]
*Lactobacillus* spp.	Acetylcholine	Primary excitatory neurotransmitter Influences synaptic plasticity and reinforces neuronal plasticity Cortical dynamics during learning and changes neuronal excitability Neurons are responsible for changing environmental conditions faster	[28,29]
*Bifidobacterium* spp.	Gamma-aminobutyric acid (GABA)	Reduces anxiety, fear, and stress	[30,31,32]
*Bifidobacterium* spp., *Bacillus* spp.	Tryptophan	Improves cognitive functions	

**Table 2:** Crude summary data on the quality of 26 papers assessed using the checklist.

	Randomized Studies		Non-Randomized Studies	
	Mean	Range	Mean	Range
**Reporting**	8.9	6–10	6.9	5–9
**External validity**	2.6	1–3	2.0	0–3
**Bias**	6.8	5–7	4.8	4–5
**Confounding**	4.5	1–6	0.9	0–2

## Data Availability

Not applicable.

## Supplementary Materials

The following supporting information can be downloaded at: https://www.mdpi.com/article/10.3390/ijms23147820/s1.
